# MiR-20a-5p suppresses tumor proliferation by targeting autophagy-related gene 7 in neuroblastoma

**DOI:** 10.1186/s12935-017-0499-2

**Published:** 2018-01-04

**Authors:** Yongbo Yu, Jie Zhang, Yaqiong Jin, Yeran Yang, Jin Shi, Feng Chen, Shujing Han, Ping Chu, Jie Lu, Huanmin Wang, Yongli Guo, Xin Ni

**Affiliations:** 1 Beijing Key Laboratory for Pediatric Diseases of Otolaryngology, Head and Neck Surgery, MOE Key Laboratory of Major Diseases in Children, Beijing Pediatric Research Institute, Beijing Children’s Hospital, Capital Medical University, National Center for Children’s Health (NCCH), Beijing, 100045 China; 2 Department of Otolaryngology, Head and Neck Surgery, Beijing Children’s Hospital, Capital Medical University, National Center for Children’s Health (NCCH), Beijing, 100045 China; 3 Department of Surgical Oncology, Beijing Children’s Hospital, Capital Medical University, National Center for Children’s Health (NCCH), Beijing, 100045 China

**Keywords:** Neuroblastoma, miR-20a-5p, Autophagy, Autophagy-related gene 7, Pediatrics

## Abstract

**Background:**

Neuroblastoma (NB) is the most common malignant tumor originating from the extracranial sympathetic nervous system in children. The molecular mechanisms underlying this disease are complex, and not completely understood.

**Methods:**

Quantitative real-time PCR (qRT-PCR) was applied to quantify the expression of miR-20a-5p and its target gene ATG7 in clinical NB tissues. The biological function of miR-20a-5p and ATG7 in SH-SY5Y cells was investigated through in vitro studies (Real-Time cell kinetic analyzer, colony formation assay, caspase-Glo 3/7 assay and western blotting). The luciferase reporter assay was conducted to verify the biological relationship between miR-20a-5p and ATG7.

**Results:**

Here we found that miR-20a-5p expression was significantly downregulated whereas its target autophagy-related gene 7 (ATG7) was increased along with clinical staging of NB progression. Correlation analysis showed that miR-20a-5p had a negative correlation trend with ATG7. In SH-SY5Y cells, forced expression of miR-20a-5p suppressed ATG7 expression, autophagy initiation and cellular proliferation while promoted apoptosis, suggesting a potential association between miR-20a-5p and ATG7. Further bioinformatic target prediction combined with protein expression and luciferase reporter assay verified that miR-20a-5p inhibited ATG7 by directly binding to its 3′-UTR, confirming the involvement of miR-20a-5p in the regulation of ATG7 in NB.

**Conclusions:**

These results clarified that miR-20a-5p inhibited cell proliferation and promoted apoptosis through negative regulation of ATG7 and thus autophagy suppression in SH-SY5Y cells. Therefore, defining the context-specific roles of autophagy in NB and regulatory mechanisms involved will be critical for developing autophagy-targeted therapeutics against NB. Both miR-20a-5p and ATG7 would be potential therapeutic targets for future NB treatment.

## Background

Neuroblastoma (NB) is the most common extracranial solid tumour occurring in children [1], which accounts for more than 15% of all pediatric oncology deaths. Pediatric patients with NB have a poor prognosis despite receiving multimodal treatments such as surgery, radiotherapy, photodynamic therapy, and chemotherapy. Half of neuroblastoma cases are classified as high-risk for disease relapse, with long-term survival less than 40% [2]. Even when whole-genome sequencing of neuroblastoma was conducted, few recurrent gene alterations (MYCN, ALK, ATRX and TERT) were identified [3–5]. The unclear pathogenesis of neuroblastoma impedes the development of therapeutic drugs discovery and effective tumor therapy. With decades of efforts, only Unituxin (dinutuximab) was approved by the US Food and Drug Administration as a novel targeted drug in 2015, which can prolong the survival of patients with high-risk NB [6]. Therefore, clear clarification of mechanisms underlying NB progression is urgently needed.

MicroRNAs (miRNAs) are small non-coding RNAs of 19–25 nucleotides in length, serving as post-transcriptional regulators of gene expression [7]. Functionally, miRNAs can regulate genes involved in diverse biological processes, such as cell proliferation, development, differentiation and apoptosis [8]. Pathologically, abnormal microRNA expression is involved in tumorigenesis [9]. In NB, miR-23a, miR-421 and miR-558 promoted tumor growth, invasion, metastasis and induced angiogenisis [10–12]. Recently, miR-451 was reported reduced in NB tissues and correlated with tumour size, lymph node metastasis, tumour-node-metastasis (TNM) stage and distant metastases [13]. These studies indicated that miRNAs contributed to diverse processes in NB, acting as oncogenes and/or tumor suppressors. The miR-20a-5p is a 23-nucleotides-length non-coding RNA. Functionally, various studies have been conducted to investigate the effects of miR-20a-5p in types of tumors. Some of the results demonstrated that miR-20a-5p promoted radio-resistance in nasopharyngeal cancer cells [14], promoted colorectal cancer invasion and metastasis [15], and repressed multi-drug resistance in osteosarcoma [16]. However, the accurate expression, function and mechanism in tumor, especially in pediatric NB, are largely unclear.

Accumulating reports have revealed that miRNAs can modulate autophagic pathways [17]. Autophagy is an intracellular process highly regulated by autophagy-related genes (ATGs) for lysosomal degradation and recycling of proteins and organelles [18]. Autophagy dysfunction can lead to severe pathological states, such as neurodegenerative diseases and particularly cancer [19, 20]. As a pivotal regulator in autophagy initiation and autophagosome formation, ATG7 contributes to tumor cell proliferation, cell death and drug resistance [21]. Recently, miR-375 was reported to inhibit autophagy and reduce viability via ATG7 in hepatocellular carcinoma cells under hypoxic conditions [22]. Moreover, miR-200b could downregulate ATG12, suppress autophagy and enhance chemosensitivity both in vivo and in vitro [23]. In pediatric NB, autophagy was also associated with chemoresistance and proliferation [24, 25], but the involved underlying mechanisms were not clear. Although evidence has linked miRNAs to autophagy, it is far from clear whether miR-20a-5p contributes to the regulatory network of autophagy in NB.

To investigate the function and underlying mechanism of miR-20a-5p in NB proliferation, this study was then conducted. We found that miR-20a-5p was significantly downregulated, while ATG7 was upregulated along with clinical staging of NB progression. MiR-20a-5p overexpression and ATG7 silence respectively inhibited cell proliferation and promoted apoptosis in SH-SY5Y cells. Furthermore, we demonstrated that miR-20a-5p negatively regulated autophagy through suppressing ATG7 by binding to its 3′-UTR. These results suggested that miR-20a-5p and ATG7-mediated autophagy might play important roles in the development and progression of NB and represent as potential therapeutic targets for NB therapy.

## Methods

### Clinical specimens and cell lines

A total of 35 fresh NB tumor specimens were collected from Beijing Children’s Hospital, Capital Medical University between May 2015 to December 2016. Informed consent was permitted from patients/guardians upon samples collection. This study was approved by the Ethics Committees of Beijing Children’s Hospital. Histologic sections were reviewed by two expert pathologists to verify the histologic diagnosis. International Neuroblastoma Staging System (INSS) is used for clinical staging of NB by clinicians. All tissues were immediately dissected, placed on ice, and stored at liquid nitrogen until use. Neuroblastoma cell line (SH-SY5Y) and human embryonic kidney cell line (293T) were purchased from Cell Resource Center, Chinese Academy of Medical Sciences (CAMS, Beijing, China). The cells were cultured in DMEM supplemented with 10% fetal bovine serum, in a humidified, 5% CO_2_ incubator at 37 °C. The SH-SY5Y was chosen as a representative cell line of NB disease and is widely used in mechanism and drug development studies in NB [26, 27]. Moreover, 293T cells were employed due to their satisfactory transfection efficiency in the luciferase reporter assay.

### RNA extraction and qRT-PCR assays

Tumor tissues were homogenized in TRIzol reagent (Invitrogen) and total RNA was extracted using Direct-zol™ RNA MiniPrep kit (Zymo Research) following manufacturer’s protocol. One microgram total RNA of each sample was reverse transcribed to cDNA in a final volume of 20 μl using RevertAid™ H Minus First Strand cDNA Synthesis Kit (Thermo Scientific). For microRNA reverse transcription, NCode™ miRNA First-Strand cDNA Synthesis kit (Invitrogen, USA) was used. The cDNA template was amplified using SYBR Green Master mix (Applied Biosystems, Thermo Fisher Scientific) and CFX96 Real-Time PCR System (Bio-rad). For human miRNA and mRNA expression detection, RNU6 and β-actin were used as reference genes. The primer sequences used were as follows: ATG7 (forward, 5′-TGCTATCCTGCCCTCTGTCTT-3′; reverse, 5′-TGCCTCCTTTCTGGTTCTTTT-3′); β-actin (forward, 5′-TGAGACCTTCAACACCCCAG-3′; reverse, 5′-GCCATCTCTTGCTCGAAGTC-3′); miR-20a-5 [forward, 5′-TAAAGTGCTTATAGTGCAGGTAG-3′; reverse, universal qPCR Primer (Invitrogen, USA)]; RNU6 (forward, 5′-CTCGCTTCGGCAGCACA-3′; reverse, 5′-AACGCTTCACGAATTTGCGT-3′).

### Cell transfection

Oligonucleotides including miR-20a-5p mimics and miR-20a-5p inhibitor were used (Thermo Scientific, USA) for overexpression or inhibition of miR-20a-5p, respectively. For cell transfection assays, the synthetic oligonucleotides were transfected into cells using a Lipofectamine RNAiMAX Kit (Invitrogen) at about 50% confluence according to the product manual. The media were changed 8 h after transfection, and the indicated cells were subjected to further investigations.

### Real-time cell proliferation assays

Real-Time cell kinetic analyzer xCELLigence RTCA (ACEA Biosciences, USA) was used to monitor dynamic changes of cell proliferation. The SH-SY5Y cells were seeded in E-plate (ACEA Biosciences, USA), and incubated at 37 °C. When the cells reached the logarithmic growth phase, the plate was detached and cells were transfected. Changes in baseline impedance resulting from the increase of cell numbers were monitored by gold micro-electrodes located at the bottom of E-plate. The proportional changes in impedance were recorded continuously and expressed as cell index (CI). The E-Plate was then incubated in the RTCA Station inside the incubator and the CI values were recorded every 20 min. Data analysis was performed using RTCA Control Unit and the preinstalled RTCA software.

### Colony formation assay

Total of 1 × 10^3^ SH-SY5Y cells were seeded in 6-well plates and transfected with oligonucleotides of mimcs or siRNA. After 10 days, cells in each well were fixed with 4% paraformaldehyde for 10 min and stained with 0.1% crystal violet for another 10 min. Cell colonies were then photographed and counted. Assays were independently conducted three times.

### Caspase-Glo 3/7 assay

For the determination of caspase-3/7 activity, the cells were seeded onto 96-well plates in triplicate, and transfected as described above. At 24 h and 48 h post-transfection, caspase-3/7 activity was determined using a Caspase-Glo 3/7 kit (Promega, USA) according to the manufacturer’s protocol. Briefly, Caspase-Glo reagent was added to each well and incubated for 1 h at room temperature. Luminescence was measured using a SpectraMax Microplate Luminometer (Molecular Devices).

### Luciferase reporter assays

To validate the predicted microRNA-binding sequence for miR-20a-5p, a fragment of the ATG7 3′-UTR containing either the predicted binding site or a mutated 3′-UTR was cloned into the GV272 vector (Genechem, Shanghai, China). After verification by DNA sequencing, the plasmids were transfected into 293T cells with or without synthetic miR-20a-5p mimic, using a Lipofectamine 2000 (Invitrogen) following the manufacturer’s instructions. Cells were collected 48 h after transfection and analyzed with the Dual-Luciferase Reporter Assay System (Promega, CA, USA). The firefly and Renilla luciferase signals were generated by SpectraMax Microplate Luminometer (Molecular Devices). Data were normalized to Renilla activity.

### RNA interference

The SH-SY5Y cells in exponential growth phase were plated at 2 × 10^5^ cells/well in 6-well plate for 24 h. Cells were then transfected with 100 nM ATG7 or scrambled siRNA using Lipofectamine RNAiMAX (Invitrogen). The siRNA oligonucleotides of ATG7 (No. 1: 5′-UCUUCGAAGUGAAGCUUCCAGAAAU-3′; No. 2: 5′-GGAGUCACAGCUCUUCCUU-3′; No. 3: 5′-CACUUCCAGUGCCUUUCCC-3′) and non-targeting control (5′-UUCUCCGAACGUGUCACGU-3′) were synthesized by Sangon Biotech, Shanghai. After transfection in antibiotic-free medium for 8 h, cells were refreshed with normal medium. Experiments were performed 72 h after transfection.

### Western blotting

After SH-SY5Y cells were transfected with mimics or siRNA, the cells were lysed in RIPA lysis buffer for 30 min. Total cellular protein was extracted and determined by the bicinchoninic acid (BCA) protein assay (Pierce, USA). Equal amounts of proteins (20 μg) were separated by SDS-PAGE, and electrophoretically transferred to PVDF membranes (Millipore, USA). After blocking in 5% nonfat milk, the membranes were incubated with the following primary antibodies overnight at 4 °C: ATG7 (CST, 1:1000), LC3 (CST, 1:1000), p62 (CST, 1:1000), GAPDH (CST, 1:1000). The secondary antibody was anti-rabbit IgG (CST, 1:2000). Subsequent visualization was performed with the ECL chemiluminescence reagent (Pierce, USA), while relative densitometric analysis was performed by Image Lab software (Bio-Rad, USA).

### Statistical analysis

The differences between groups were analyzed by t test or one-way analysis of variance. All data were analyzed by using the SPSS 16.0 and JMP 12.0 software. The relationship between miR-20a-5p and ATG7 mRNA was analyzed by correlation and linear regression analysis. *p* < 0.05 was considered to be statistically significant.

## Results

### Clinic-pathological characteristics of enrolled patients

A total of 35 diagnosed NB patients were recruited into our study. The summary of the clinic-pathological characteristics is detailed in Table 1, including age at diagnosis, gender, and tumor stage. More than 70% patients were less than 5 years old. According to the INSS, four patients were classified as stage I, three as stage II, nine as stage III, and nineteen as stage IV.

**Table 1:** Clinical characteristics of NB patients enrolled

Parameters	Classes	Number of patients (%)
Age of diagnosis	Total	35
	0–5	26 (74.3%)
	5–10	9 (25.7%)
Gender	Total	35
	Male	13 (37.1%)
	Female	22 (62.9%)
Tumor stage	Total	35
	I	4 (11.4%)
	II	3 (8.6%)
	III	9 (25.7%)
	IV	19 (54.3%)

### Both miR-20a-5p and ATG7 was dysregulated in NB samples and correlated with tumor stage

**Fig. 1:**
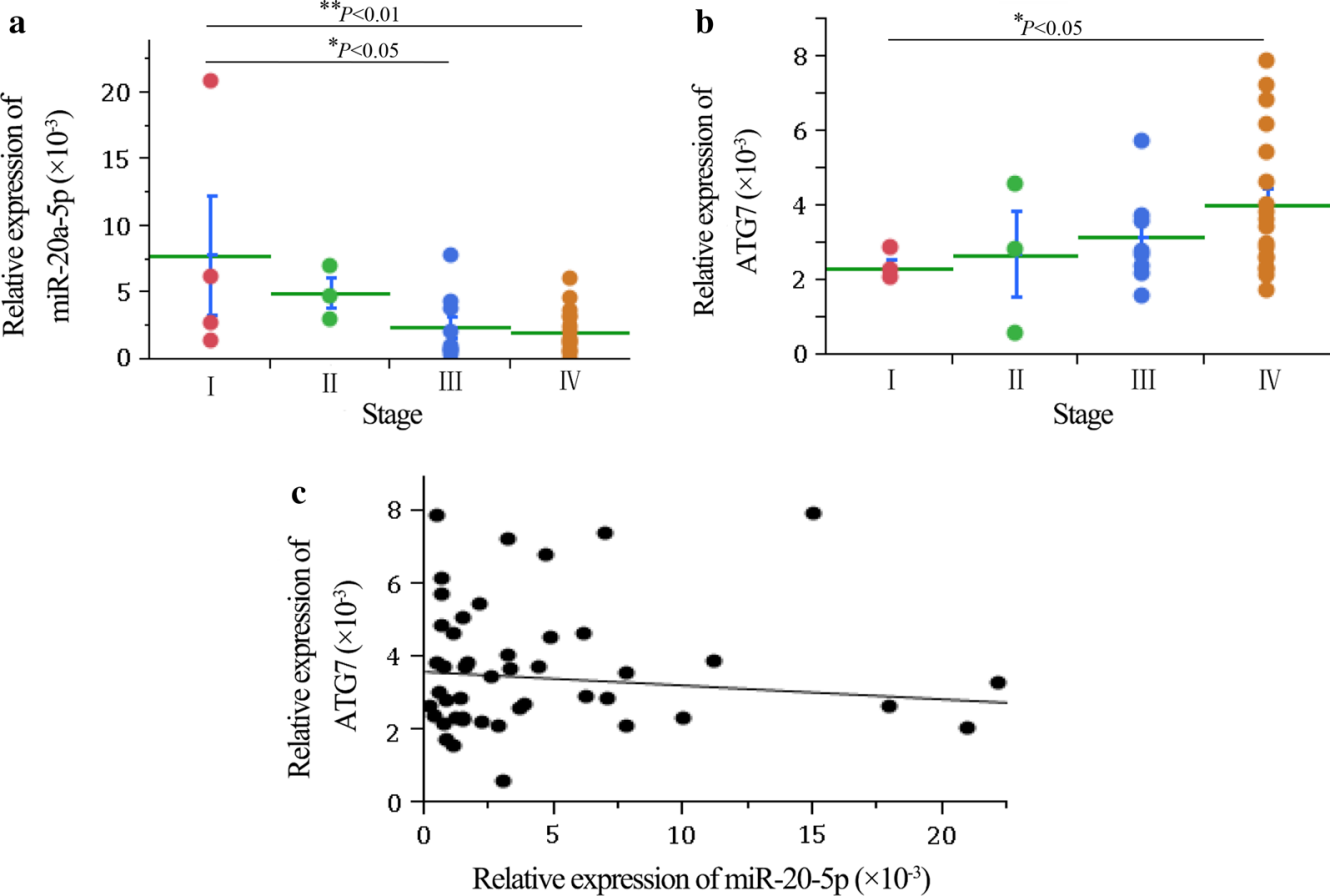
Relative expression of miR-20a-5p and ATG7 in clinical NB tissues. **a** miR-20a-5p is downregulated while **b** ATG7 is upregulated in high staging samples compared with those in low staging samples. **c** Correlation analysis showed that miR-20a-5p had a negative correlation trend with ATG7 (r = − 0.04; *p* = 0.632)

To investigate the role of miR-20a-5p and ATG7 in NB progression, quantified expression was detected in 35 NB specimens by RT-PCR. We found that the expression levels of miR-20a-5p in tumors of stage I was significantly higher than those in stage III and stage IV (Fig. 1a). However, ATG7 expression was markedly upregulated (Fig. 1b) in high staging samples compared with those in low staging samples. These results indicated that miR-20a-5p and ATG7 were significantly associated with NB stage. Moreover, correlation analysis showed that miR-20a-5p level had a negative correlation trend with ATG7 (Fig. 1c) (r = − 0.04; p = 0.632), suggesting that miR-20a-5p might involve in the regulation of ATG7.

### ATG7 was a direct target of miR-20a-5p in SH-SY5Y cells

**Fig. 2:**
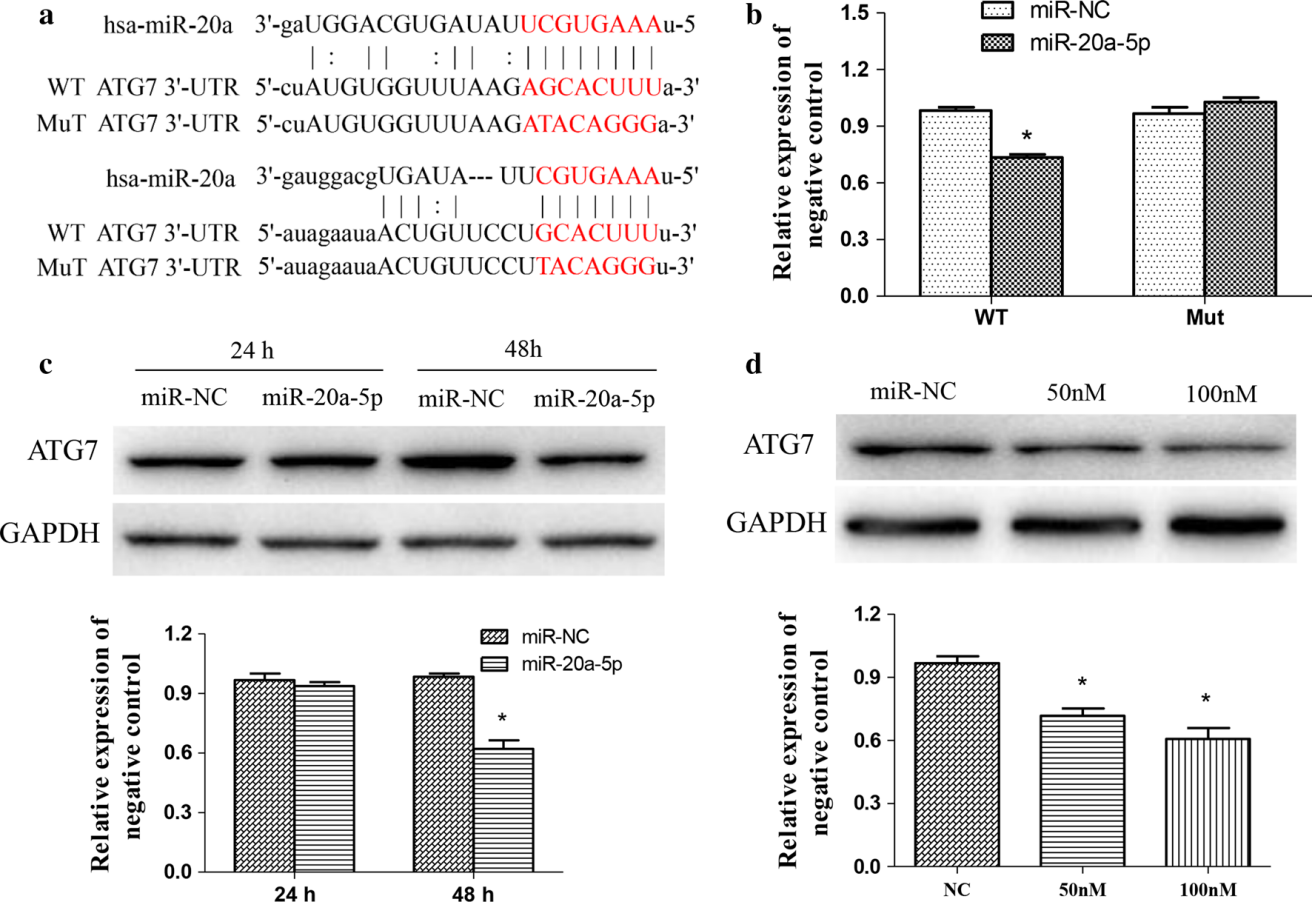
ATG7 was a direct target of miR-20a-5p in SH-SY5Y cells. **a** Schematic putative target sites of miR-20a-5p in 3′-UTR of ATG7, and the sequence of miR-20a-5p mutant (performed as mut). **b** Luciferase assay of wild type (WT) or mutated (Mut) ATG7 3′-UTR reporter co-transfected with miR-20a-5p or the control for 48 h. **c** Western blotting analysis of ATG7 protein levels in SH-SY5Y cells treated with miR-20a-5p mimics at indicated time and **d** concentration. The relative densitometric analysis of the protein bands were performed. *p < 0.05 vs. control. Data are expressed as mean ± SD, n = 3. miR-NC, negative control microRNA

To identify the mRNA targets of miR-20a-5p, we performed a bioinformatics analysis using the publicly available algorithm (TargetScan 6.2) and found that miR-20a-5p could target and regulate ATG7 translation. To confirm the prediction, we conducted luciferase reporter assay after transfection of ATG7 3′-UTR with wild type (Wt) or mutant (Mut) into 293T cells (Fig. 2a), along with miR-20a-5p mimics or controls. As shown in Fig. 2b, miR-20a-5p reduced luciferase activity in cells transfected with Wt ATG7 3′-UTR, but no effects in cells transfected with Mut ATG7 3′-UTR (Fig. 2b). Additionally, western blotting analysis showed that miR-20a-5p dramatically suppressed ATG7 protein levels in dose- and time-dependent manners (Fig. 2c, d). These collective results suggest that miR-20a-5p negatively regulates ATG7 gene expression by directly binding to 3′-UTR of ATG7.

### MiR-20a-5p overexpression and ATG7 knockdown suppressed proliferation and progression in SH-SY5Y cells

**Fig. 3:**
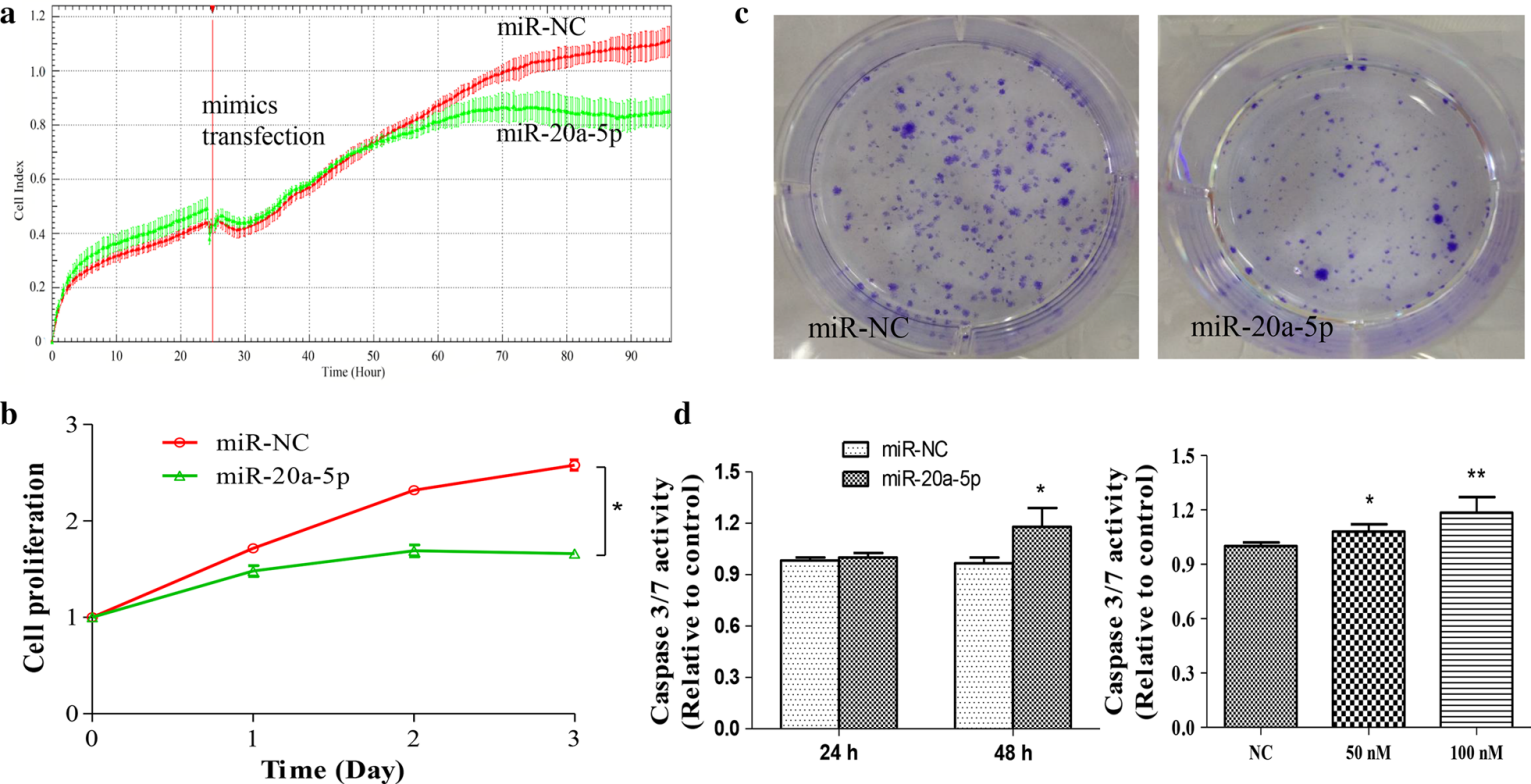
MiR-20a-5p inhibited cell proliferation and promotes cellular apoptosis in SH-SY5Y cells. **a** Effects of miR-20a-5p overexpression on cell proliferation measured by Real-Time cell kinetic analyzer (RTCA) and **b** quantification. **c** Representative photographs of colonies formed in 14 days after mimics transfection. **d** Caspase-3/7 activity in SH-SY5Y cells treated with miR-20a-5p mimics at indicated time and concentration. Data are expressed as mean ± SD, n = 3. *p < 0.05 vs. control, Student’s t test. miR-NC, negative control microRNA

To investigate biological function of miR-20a-5p in proliferation of neuroblastoma, miR-20a-5p mimics were transfected into SH-SY5Y cells and RTCA device was used to monitor cell index in real time (Fig. 3a). Quantitative analysis result showed that miR-20a-5p overexpression significantly inhibited the proliferation activity of SH-SY5Y cells (Fig. 3b). Moreover, colony formation assay further revealed that miR-20a-5p markedly suppressed SH-SY5Y cell growth, as indicated by reduction of colony numbers and intensity (Fig. 3c). These results indicated that miR-20a-5p overexpression suppressed proliferation and progression of SH-SY5Y cells.

**Fig. 4:**
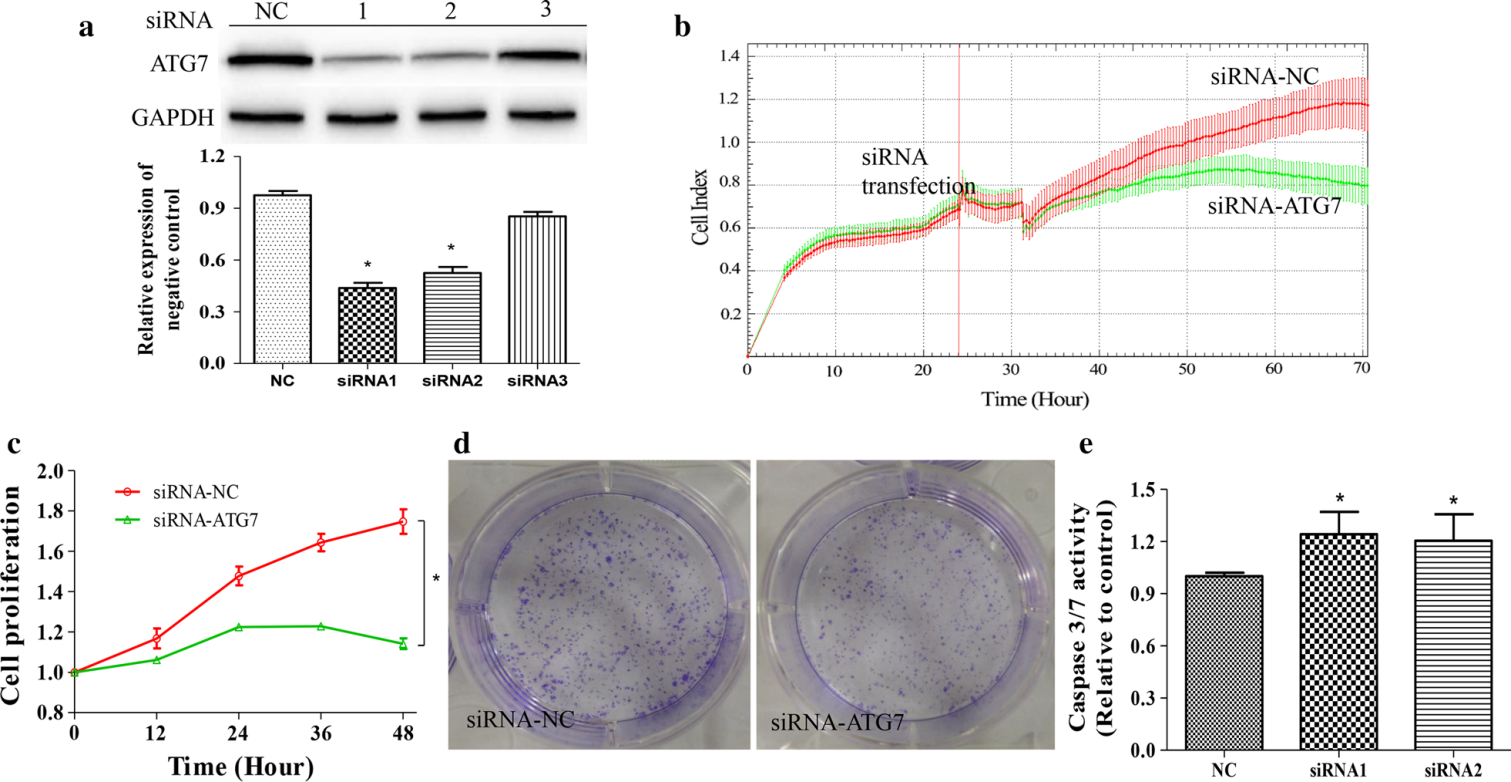
ATG7 inhibited cell proliferation and promotes cellular apoptosis in SH-SY5Y cells. **a** Western blotting validation of siRNAs on ATG7 knockdown. **b** Effects of ATG7 knockdown on cell proliferation measured by Real-Time cell kinetic analyzer (RTCA) and **c** quantification. **d** Representative photographs of colonies formed in 10 days after siRNA1 transfection. **e** Caspase-3/7 activity in SH-SY5Y cells transfected with ATG7 siRNA1 and siRNA2. Data are expressed as mean ± SD, n = 3. *p < 0.05 vs. control, Student’s t test. siRNA-NC, negative control siRNA

To evaluate the effects of ATG7 on SH-SY5Y cells progression, we suppressed endogenous ATG7 expression by specific siRNAs. As shown in Fig. 4a, ATG7-targeted siRNA effectively silenced ATG7 expression (Fig. 4a), and significantly inhibited proliferation of SH-SY5Y cells both in RTCA real-time detection and quantitative analysis (Fig. 4b, c). The colony formation assay indicated that ATG7 knockdown suppressed colony forming ability in SH-SY5Y cells (Fig. 4d). These results indicated that ATG7 knockdown suppressed proliferation and progression in SH-SY5Y cells.

### MiR-20a-5p overexpression and ATG7 knockdown suppressed autophagy in SH-SY5Y cells

**Fig. 5:**
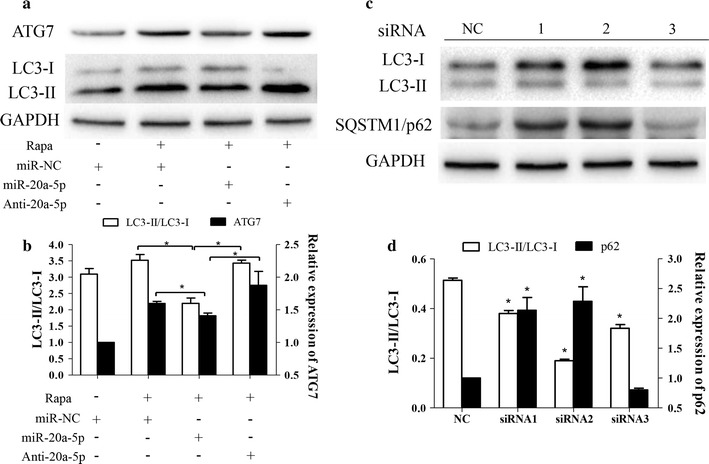
Effects of miR-20a-5p and ATG7 on autophagy in SH-SY5Y cells. **a** Western blotting validation of ATG7 knockdown on autophagy suppression and **b** relative densitometric analysis of the protein bands. **c** Forced miR-20a-5p expression inhibited ATG7 expression and suppressed rapamycin-induced autophagy, while miR-20a-5p inhibitor reversed these effects. **d** Relative densitometric analysis of the protein bands. *p < 0.05 vs. indicated groups. Data are expressed as mean ± SD, n = 3. Rapa, rapamycin; miR-NC, negative control microRNA

To investigate the suppression of miR-20a-5p on autophagy, rapamycin was applied to establish autophagy model in SH-SY5Y cells. In circumstances of autophagy induced by rapamycin (Fig. 5a), relative densitometric analysis showed that miR-20a-5p inhibited autophagy marker of LC3-II/LC3-I ratio and ATG7 expression. In addition, miR-20a-5p inhibitor significantly reversed these effects. Thus miR-20a-5p was demonstrated to suppress autophagy and ATG7 expression (Fig. 5b). Since ATG7 was identified as a direct target of miR-20a-5p in luciferase reporter assay, these results clarified that ATG7 potentially acted as an executor underlying miR-20a-5p-mediated autophagy suppression.

Since ATG7 plays an essential role in complete autophagy, the effects of ATG7 knockdown on autophagy inhibition was further verified by measuring LC3-II/LC3-I ratio (autophagy marker) and autophagy substrate SQSTM1/p62. As shown in western blotting (Fig. 5c), all the ATG7 specific siRNAs increased LC3-I levels, suggesting that the conversion of LC3-I to LC3-II was inhibited. The elevated LC3-I thus accounts for significantly decrease of LC3-II/LC3-I ratio (Fig. 5d). Since p62 is a substrate for autophagy degradation, increased p62 expression (Fig. 5c) by ATG7-targeted siRNA confirmed that ATG7 knockdown suppressed cellular autophagy. These results validated that ATG7 mediated autophagy activation in SH-SY5Y cells.

### MiR-20a-5p overexpression and ATG7 knockdown increased caspase-3/7 activity

To determine the effects of miR-20a-5p and ATG7 on cellular apoptosis, caspase-3/7 activity in SH-SY5Y cells were examined post-transfection with miR-20a-5p mimics and ATG7 siRNA, respectively. The results showed that caspase-3/7 activity was significantly increased in SH-SY5Y cells transfected with miR-20a-5p mimics in a time- and dose-dependent manner, as compared with the negative control (Fig. 3d). Similarly, ATG7 siRNA significantly enhanced the activity of caspase-3/7 in SH-SY5Y cells (Fig. 4e). These results suggested that miR-20a-5p overexpression and ATG7 knockdown promoted cellular apoptosis, respectively.

## Discussion

**Fig. 6:**
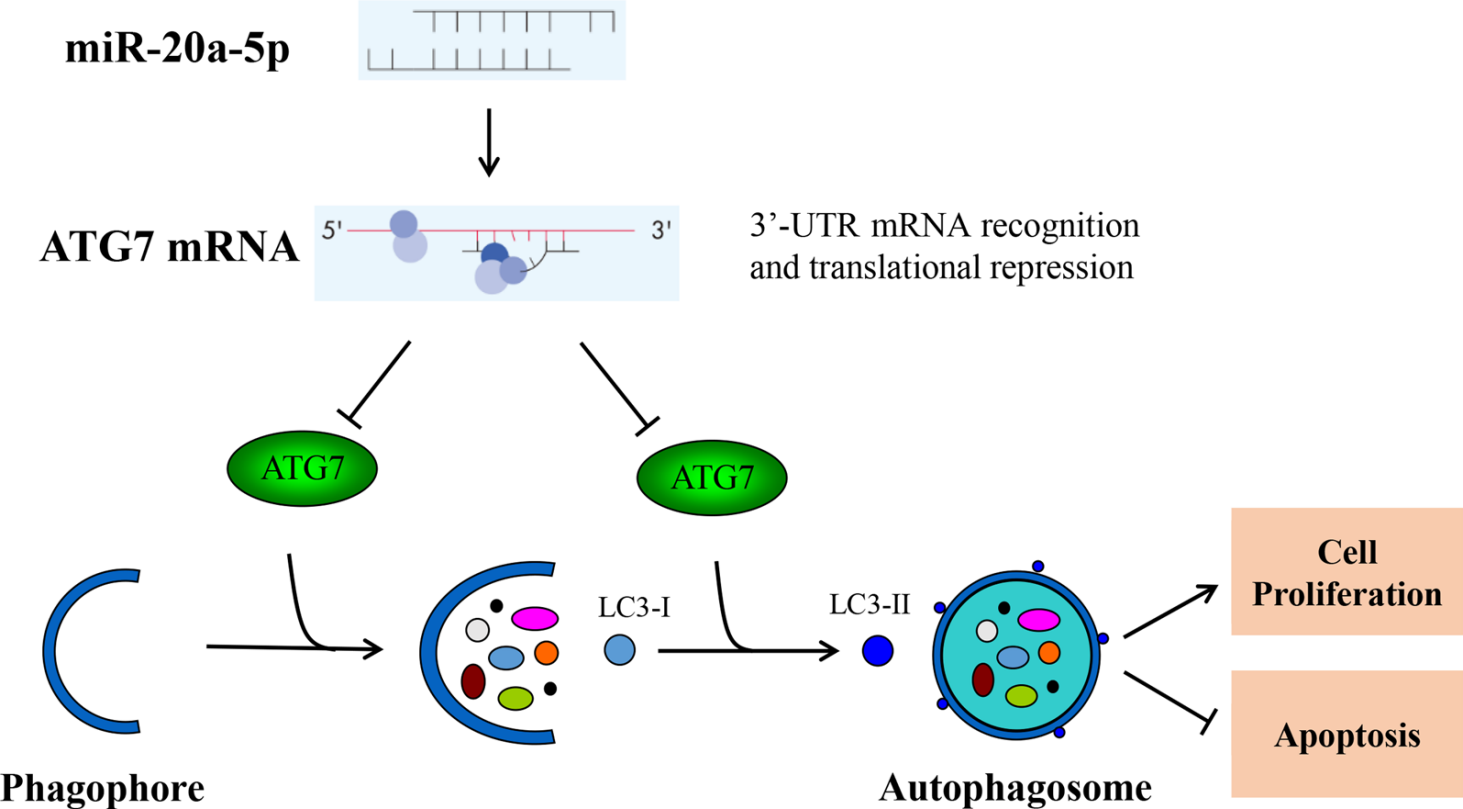
The putative schematic representation of pathway involved in miR-20a-5p-mediated suppression of cell proliferation in SH-SY5Y cells. MiR-20a-5p recognizes and binds to 3′-UTR of ATG7, repressing ATG7 gene translation. Since ATG7 contribute to autophagosome initiation and completion, which accounts for cell proliferation and anti-apoptosis, miR-20a-5p-mediated ATG7 inhibition thus results in cell proliferation suppression and further apoptosis

Neuroblastoma (NB) is the most common extracranial solid tumor in children. Despite receiving multimodal treatment, high-risk NB remains a clinical challenge with survival rates below 50% [2]. Even though genetic alterations have been identified, such as MYCN, ALK, ATRX and TERT [3–5], mechanisms underlying NB progression were still not clear. In the present study, we found that miR-20a-5p was downregulated while ATG7 was upregulated in NB tumor specimens. Additional bioinformatics analysis suggested that miR-20a-5p could bind to 3′-UTR of ATG7 and suppress ATG7 translation. Further evidence confirmed that miR-20a-5p inhibited cell proliferation and promoted apoptosis through ATG7-mediated autophagy in SH-SY5Y cells (Fig. 6).

MiRNAs are involved in the etiology, progression, and prognosis of cancer [7, 9]. For tumorigenesis, miRNAs produce competing oncogenic and tumor suppressive effects by suppressing both tumor suppressive mRNAs and oncogenic mRNAs, respectively [9, 28, 29]. To verify the effects of miR-20a-5p on NB tumorigenesis, in vitro experiments were performed in this study, demonstrating that miR-20a-5p inhibited cell proliferation and colony formation. Consistent with our findings that miR-20a-5p was downregulated in pediatric NB, miR-451 expression was reported reduced in NB specimens, and correlated with tumour size, lymph node and distant metastasis, and tumour-node-metastasis (TNM) stage [13].

Functionally, miRNAs regulate gene expression by suppressing target mRNA translation and reducing mRNA stability. Herein, autophagy-related gene 7 (ATG7) was a bioinformatics target of miR-20a-5p, and luciferase assay validated miR-binding sequence for miR-20a-5p. Western blotting analysis further showed that forced expression of miR-20a-5p dramatically suppressed ATG7 protein levels in a dose- and time-dependent manner. Since ATG7 is crucial in autophagy activation and autophagosome formation [30], these results suggest that miR-20a-5p may inhibit cell proliferation through autophagy via negative regulation of ATG7 in SH-SY5Y cells. Similarly, miR-337-3p was reported to suppress NB progression by repressing the transcription of matrix metalloproteinase 14 [31]. However, further study was needed to clarify that ATG7-mediated autophagy contributed to NB progression.

The degradation process of autophagy is controlled by autophagy-related genes (ATGs), including ATG7 [32]. Normally, autophagy plays an essential role in maintaining cellular homeostasis and physiological functions. However, autophagy confers stress tolerance as a carcinogenetic effect, which promote tumor development, malignant transformation and drug or apoptosis resistance [21, 33, 34]. In clinical NB tissues, we found that ATG7 was upregulated. In an in vitro model, ATG7 knockdown significantly inhibited cellular proliferation and colony formation as well, suggesting that ATG7-mediated autophagy may be involved in cell proliferation and tumor growth of pediatric NB. Consistent with our findings, ATG7 knockout can significantly reduce tumor cell tumorigenicity and promote the transformation of lung cancer into benign tumor [35]. In addition, ATG7 deficiency can completely inhibit tumor occurrence and development of intestinal epithelial cells in mice [36]. Herein, ATG7 knockdown was further validated to inhibit autophagy induction and substrate degradation of SQSTM1/p62. Taken all these results into consideration, ATG7-mediated autophagy is was postulated to contribute to NB progression.

In the present study, miR-20a-5p was demonstrated to suppress ATG7 expression and autophagy, while miR-20a-5p inhibitor reversed these effects. Such evidences indicated the involvement of miR-20a-5p in autophagy. Previously, miRNAs were reported to account for the regulation of autophagy, including autophagy initiation, autophagy vesicles formation and substrates degradation [17]. Numerous miRNAs regulate autophagy by suppressing autophagy-related proteins, such as miR-17, miR-20a, miR-106b and miR-375 [22, 37, 38]. Wu et al. reported that miR-20a and miR-106b negatively regulate starvation-induced autophagy by suppressing ULK1 in C2C12 myoblasts [39]. Due to the fact that ATG7 can simultaneously regulate two ubiquitin-like systems of ATG12 and ATG8 in autophagy activation and autophagosome formation [30], the miR-20a-5p-mediated silence of ATG7 indicated that miR-20a-5p contributed to autophagy inhibition.

There also exists crosstalk between autophagy and apoptosis even though the details of molecular switching points are still not fully elucidated [40, 41]. Autophagy inhibition by ATG7 silence significantly enhanced the activity of caspase-3/7, suggesting that suppression of autophagy promotes cellular apoptosis. In line with our results, knockdown of essential autophagy genes in tumor cells could also potentiate the induction of cell death. In human pancreatic cancer, inhibition of autophagy led to tumor regression and extended survival in xenografts and genetic mouse models [42]. In the present study, miR-20a-5p suppressed autophagy and ATG7 expression and enhanced cellular apoptosis, indicating that autophagy might contribute to cell proliferation and apoptosis resistance in SH-SY5Y cells. The putative molecular mechanism underlying miR-20a-5p-mediated effects in SH-SY5Y cells is presented in the schematic diagram. This study improved our understanding that miRNAs-mediated autophagy was involved in the regulation of NB development and may provide potential new therapeutic targets for the management of pediatric NB.

## Conclusions

In summary, we demonstrated that miR-20a-5p was significantly downregulated while ATG7 was upregulated along with clinical staging of NB progression. Both miR-20a-5p overexpression and ATG7 silence impaired cell growth, colony formation and promoted apoptosis in SH-SY5Y cells in vitro. Furthermore, we confirmed that miR-20a-5p negatively regulated autophagy through suppressing ATG7 by binding to its 3′-UTR. This study extends our knowledge about ATG7 regulation by miRNAs, and suggests that both miR-20a-5p and ATG7 may be of potential value as novel therapeutic targets for pediatric NB.

## Abbreviations

NB: neuroblastoma
ATG7: autophagy-related gene 7
UTR: untranslated region
miRNA: microRNA
FBS: fetal bovine serum
RNU6B: U6B small nuclear RNA
